# Virtuelle Histopathologie des Pankreas: 3D-Einblicke mittels synchrotronbasierter Bildgebung

**DOI:** 10.1007/s00292-025-01533-8

**Published:** 2026-01-08

**Authors:** Matthias Martin Gaida, Lukas Hessel, Caroline Victoria Schimmel, Klara Schulze, Verena Wagner, Philipp Mayer, Jonas Albers, Elizabeth Duke, Martin Loos, Gabriel Alexander Salg

**Affiliations:** 1 Institut für Pathologie, Universitätsmedizin der Johannes Gutenberg-Universität Mainz, Mainz, Deutschland; 2 Forschungszentrum Immuntherapie, Universitätsmedizin der Johannes Gutenberg-Universität Mainz, Mainz, Deutschland; 3 Joint Unit Immunpathologie, Translationale Onkologie, Institut für Pathologie, Universitätsmedizin der Johannes Gutenberg-Universität Mainz, Mainz, Deutschland; 4 European Molecular Biology Laboratory (EMBL), Hamburg, Deutschland; 5 Klinik für Allgemein‑, Viszeral- und Transplantationschirurgie, Universitätsklinikum Heidelberg, Im Neuenheimer Feld 420, 69120 Heidelberg, Deutschland; 6 Klinik für Diagnostische und Interventionelle Radiologie, Universitätsklinikum Heidelberg, Heidelberg, Deutschland; 7 Institut für Diagnostische Radiologie und Neuroradiologie, Universitätsklinikum Greifswald, Greifswald, Deutschland; 8 Medizinische Fakultät, Universität Heidelberg, Heidelberg, Deutschland

**Keywords:** Virtuelle Pathologie, Pankreaskarzinom, Duktales Adenokarzinom, Röntgenbildgebung, Neuroendokriner Tumor der Bauchspeicheldrüse, Virtual pathology, Pancreatic cancer, Ductal adenocarcinoma, X-ray imaging, Pancreatic neuroendocrine tumor

## Abstract

**Hintergrund:**

Die konventionelle histopathologische Diagnostik stößt bei der Beurteilung komplexer, dreidimensionaler Gewebearchitekturen an inhärente methodische Grenzen. Insbesondere bei heterogen zusammengesetzten Geweben wie dem Pankreas oder bei komplexen Gewebspathologien erschwert die Beschränkung auf zweidimensionale Schnittbilder die ubiquitäre Erfassung morphologischer Merkmale.

**Ziel der Arbeit (Fragestellung):**

Ziel dieser Studie ist es, das Potenzial der synchrotronbasierten Phasenkontrastbildgebung (SRµCT) für die hochauflösende, dreidimensionale Visualisierung verschiedener Pankreasgewebe zu demonstrieren. Anhand dreier paradigmatischer Fallbeispiele werden morphologische Parameter volumetrisch erfasst und mit korrespondierenden immunhistochemischen Markerprofilen korreliert.

**Material und Methoden:**

Gewebestanzen aus formalinfixierten, paraffineingebetteten Blöcken von humanen Pankreasgewebeproben wurden mittels SRµCT volumetrisch erfasst. Das untersuchte Probenmaterial wurde als Microarrays weiterverarbeitet. Konsekutive Schnitte und immunhistochemische Färbungen wurden mit den 3D-Datensätzen korreliert.

**Ergebnisse:**

Die Bildgebung ermöglichte die differenzierte räumliche Darstellung funktioneller Kompartimente und neoplastischer Infiltrationsmuster. Nichtneoplastisches Gewebe zeigte klar abgegrenzte Kompartimente. Ein gut differenzierter neuroendokriner Tumor präsentierte trabekuläre Binnenstrukturen. Das duktale Adenokarzinom zeigte ein infiltratives Wachstumsmuster mit diffuser, heterogener Architektur, irregulären Gangformationen und Stromadesmoplasie. Die virtuelle Schnittführung ermöglichte die Analyse in jeder Raumrichtung. Durch Korrelation mit immunhistochemischen Markerprofilen konnten morphofunktionelle Merkmale validiert werden.

**Schlussfolgerung:**

Die SRµCT ist eine hochsensitive Methode, die nichtinvasiv und ohne Färbung dreidimensionale Einsichten in die Gewebearchitektur des Pankreas unter Nutzung archivierter Paraffinblöcke erlaubt. Die Methode bietet neue Perspektiven für Forschung, Lehre und potenziell erweiterte Spezialdiagnostik.

## Abstract (en)

**Background:**

Conventional histopathology faces methodological limitations when assessing complex three-dimensional tissue architectures. In particular, for heterogeneous tissues such as the pancreas or in complex tissue pathologies, restriction to two-dimensional sections hampers comprehensive recognition of morphological features.

**Objective:**

This study aims to demonstrate the potential of synchrotron-based phase-contrast imaging (SRµCT) as a tool for high-resolution visualization of pancreatic tissue. Three representative case examples were analyzed to capture morphological parameters volumetrically and correlate them with immunohistochemical marker profiles.

**Materials and methods:**

Tissue cores from formalin-fixed, paraffin-embedded human pancreatic samples were volumetrically assessed using SRµCT. The investigated material was further processed as microarrays. Serial sections and immunohistochemical stains were correlated with the 3D datasets.

**Results:**

SRµCT enabled detailed spatial visualization of functional compartments and neoplastic infiltration patterns. Non-neoplastic tissue revealed distinct morphological compartments. A well-differentiated neuroendocrine tumor exhibited trabecular architecture, whereas ductal adenocarcinoma displayed infiltrative growth with diffuse, heterogeneous architecture, irregular duct formations and stromal desmoplasia. Virtual slicing permitted orientation-independent analyses. Correlation with immunohistochemical profiles validated the morphofunctional findings.

**Conclusion:**

SRµCT is a sensitive, non-invasive technique providing label-free 3D insights into pancreatic architecture. It opens new perspectives for research, teaching, and potentially advanced diagnostic applications.

## Hintergrund und Fragestellung

### Bedarf an dreidimensionalen, hochauflösenden Verfahren in der Pathologie

Die histologische Architektur biologischen Gewebes ist intrinsisch dreidimensional. Dennoch basiert die histopathologische Diagnostik nach wie vor maßgeblich auf der zweidimensionalen Analyse dünner Gewebeschnitte, typischerweise in standardisierten Schnittebenen. Diese Methode ist der diagnostische Goldstandard bei einer Vielzahl von Erkrankungen. Insbesondere im wissenschaftlichen, jedoch auch didaktischen Kontext, bringt die klassische Methode Limitationen mit sich: Die räumliche Organisation wird nur fragmentiert erfasst und relevante pathologische Muster können durch die Wahl der Schnittebene, mechanische bzw. technische Artefakte oder unvollständige Repräsentation teilweise erschwert beurteilbar sein [1–3]. So können im zweidimensionalen Schnitt getrennt liegende Tumorfoci einem verzweigten, aber zusammenhängenden Netzwerk in der dreidimensionalen Betrachtung entsprechen [4]. Daneben ist davon auszugehen, dass spezielle, mitunter prognostisch relevante Tumoreigenschaften wie tumoröse Gefäß- oder Perineuralscheideninvasion in ihrer Häufigkeit im konventionellen Schnitt unterschätzt oder unter Umständen nicht repräsentiert werden [1, 5, 6]. Vor diesem Hintergrund gewinnen hochauflösende 3D-Bildgebungsverfahren zunehmend an Bedeutung [1–3, 7]. Sie ermöglichen eine umfassende Visualisierung der Gewebearchitektur im Raum. Die synchrotronbasierte Phasenkontrastbildgebung („synchrotron radiation micro-computed tomography“, SRµCT) stellt hierbei eine besonders leistungsfähige Methode dar, die eine dreidimensionale Erfassung von biologischen Gewebeproben in subzellulärer Auflösung erlaubt, ohne die Notwendigkeit mechanischer Schnitte oder exogener Kontrastmittel.

### Technischer Hintergrund: Synchrotronbildgebung für biologisches Gewebe

Konventionelle Röntgenbildgebung, wie sie in der klassischen Mikro-Computertomographie (µCT) eingesetzt wird, basiert auf Absorptionskontrasten. Der geringe Dichtekontrast besonders zwischen Weichgewebekomponenten macht lange Belichtungszeiten oder Kontrastmittel erforderlich [8–10]. Die SRµCT überwindet diese Limitationen durch den Einsatz von Phasenkontrasttechnologie, welche Unterschiede im Brechungsindex an Strukturübergängen im Gewebe ausnutzt [9, 11]. Synchrotrone erzeugen eine kohärente, hochenergetische und stark gebündelte Röntgenstrahlung mit hoher Brillanz, die eine bildgebende Auflösung im Submikrometerbereich ermöglicht. Diese physikalischen Eigenschaften ermöglichen die kontrastreiche, zerstörungsfreie Darstellung selbst feinster Strukturen in Weichgewebe [9]. Die resultierenden 3D-Datensätze sind isotrop, virtuell rotierbar und in beliebiger Ebene durchschneidbar. Auf diese Weise kann ein virtuelles, mikroskopisches Volumenmodell erzeugt werden, das mit klassischer Histologie korreliert und damit um zusätzliche Information erweitert werden kann.

### Virtuelle 3D-Histopathologie des Pankreas

In dieser Arbeit wird die Anwendung der SRµCT auf formalinfixierte, paraffineingebettete Pankreasproben mit subzellulärer Auflösung präsentiert. Ziel ist es, die morphologischen Gewebecharakteristika im gesunden und erkrankten Gewebe zu analysieren und mit klassischen histologischen und immunhistochemischen Färbungen zu korrelieren. Es werden exemplarische Datensätze von Pankreasgewebe von 3 Patient:innen vorgestellt: nichtneoplastisches, pathologisch nicht alteriertes Pankreasgewebe als Referenzstruktur, ein gut differenzierter neuroendokriner Tumor (PanNET) sowie ein duktales Adenokarzinom des Pankreas (PDAC). Es existieren bereits Vorarbeiten, in welchen SRµCT für die Untersuchung von Pankreasgewebe aus Maus, Ratte, Schwein und Mensch verwendet wurde [7, 12–14]. Hierbei wurden endokrine und exokrine Organanteile und deren pathologische Veränderungen sowie Gefäßnetzwerke exemplarisch untersucht [7, 12–14]. Durch die Methodik des SRµCT konnte hier unter anderem ein krankheitsspezifischer Strukturverlust des Pankreasgewebes untersucht werden.

## Material und Methoden

### Probenmaterial

Untersucht wurden formalinfixierte, paraffineingebettete (FFPE) Gewebeproben des menschlichen Pankreas aus der Pankreasbiobank des Europäischen Pankreaszentrums am Universitätsklinikum Heidelberg. Pathologisch verändertes Pankreasgewebe wurde dabei im Rahmen chirurgischer Resektionen gewonnen. Normalgewebe wurde aus Pankreata von Organspendern gewonnen, welche nicht für eine Transplantation geeignet waren.

### Vorbereitung der Proben

**Abb. 1:**
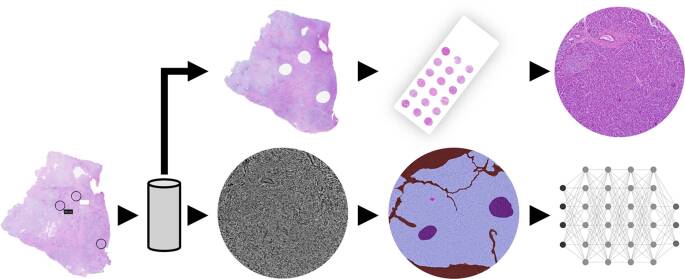
Schematische Darstellung der Probenverarbeitung und Datenerhebung. Basierend auf Scans HE-gefärbter Schnitte von Gewebeblöcken aus der Pankreasbiobank wurden durch einen Pathologen Zielregionen bestimmt, aus welchen die Stanzbiopsien entnommen wurden. Die korrekte Entnahmelokalisation wurde erneut histologisch verifiziert. Die Stanzen wurden mittels synchrotronbasierter Phasenkontrastbildgebung (SRµCT) volumetrisch erfasst und erneut in Gewebe-Microarrays eingebettet. Der 3D-Datensatz konnte nun analysiert bzw. annotiert werden. Parallel wurden aus den Gewebe-Microarrays erneut Schnitte und konsekutive immunhistochemische Färbungen angefertigt. Die entstehenden Daten können korreliert und beispielsweise mithilfe von künstlicher Intelligenz ausgewertet werden

Von allen FFPE-Blöcken wurde ein Gewebeschnitt entnommen und mittels Hämatoxilin-Eosin (HE) gefärbt (Abb. 1). Die ursprüngliche pathologische Diagnose wurde erneut fachärztlich gemäß aktueller WHO-Klassifikation bestätigt und mindestens eine Zielregion ausgewählt. Aus diesen Zielregionen wurden Biopsiestanzen mit einem Durchmesser von 1,0 mm entnommen. Die Stanze wurde auf einer 3D-gedruckten Vorrichtung in magnetischen Goniometer-Basen (B5 Goniometer Base, Jena Bioscience GmbH, Jena, Deutschland) fixiert [9]. Von allen biopsierten FFPE-Blöcken wurde erneut ein Gewebeschnitt entnommen und gefärbt (HE), um die korrekte Entnahmeregion zu verifizieren.

### SRµCT-Durchführung und Datenverarbeitung

Die 3D-Bildgebung erfolgte an der EMBL-Beamline P14 an der PETRA III Speicherring-Röntgenstrahlungsquelle des Deutschen Elektronensynchrotrons (DESY), Hamburg, Deutschland. Verwendet wurde eine Synchrotronstrahlung mit einer Energie von 12,7–18,0 keV im propagationsbasierten Phasenkontrastmodus. Hinsichtlich detaillierter Beschreibung des Messaufbaus und Etablierung der Datenerhebung an biologischem Gewebe verweisen wir auf die Arbeit von Albers et al. [9]. Die maximal erreichbare Auflösung beträgt 0,325 µm Pixelgröße in einem resultierenden Volumen (Field of View, FOV) von 666 µm × 666 µm. In dieser Studie wurden pro Probe 1810 Projektionen über 181° aufgenommen (t_exp_ = 10 ms). Die effektive Voxelgröße betrug 660 nm bei einem FOV von 1,35 mm. Die Messungen wurden bei Raumtemperatur durchgeführt. Der Prozess von Beginn der Messung bis zur Vervollständigung von automatisierter 3D- und Phasenrekonstruktion benötigte ca. 3 min pro FOV. Die SRµCT-Rohdaten wurden mit standardisierten Algorithmen (Flat-Field-Korrektur, Phasenrückgewinnung) vorverarbeitet und mittels Grid-REc rekonstruiert [15, 16]. Die Nachbearbeitung und Analyse der 3D-Datensätze erfolgte mit Dragonfly (v2024.1, COMET Technologies Canada Inc., Montreal, Kanada) und VG Studio Max (v3.4.3, Volume Graphics GmbH, Heidelberg, Deutschland). Für Segmentierungen wurden kombinierte manuelle und halbautomatisierte Ansätze verwendet.

### Weiterverarbeitung der Proben, immunhistochemische Untersuchung

Die Biopsiestanzen wurden in Gewebe-Microarrays weiterverarbeitet. Hieraus wurden klassische Paraffinschnitte (4,0 µm) seriell generiert. Neben standardisierten HE-Färbungen wurden immunhistochemische Färbungen gegen Insulin (Verdünnung 1:100, „monoclonal mouse anti-human“, #sc-8033 Lot F1721, Santa Cruz Biotechnology Inc., Dallas, TX, USA), Ki-67 (Verdünnung 1:200, „monoclonal rabbit anti-human“, #12202 Lot 8, Cell Signaling Technology, Danvers, MA, USA), Vimentin (Verdünnung 1:800, „monoclonal rabbit anti-human“, #5741 Lot 12, Cell Signaling Technology, Danvers, MA, USA) und E‑Cadherin (Verdünnung 1:400, „monoclonal rabbit anti-human“, #3195 Lot 15, Cell Signaling Technology, Danvers, MA, USA) durchgeführt. Die gefärbten Gewebeschnitte wurden mit 40facher Vergrößerung mit dem NanoZoomer S60 (Hamamatsu Photonics, Hamamatsu City, Japan) als Ganzschnittbilder digitalisiert. Eine Korrelation mit den SRµCT-Daten erfolgte manuell.

## Ergebnisse

Die synchrotronbasierte Phasenkontrastbildgebung (SRµCT) stellte die pankreatische Gewebearchitektur in allen 3 Raumdimensionen mit isotroper Auflösung dar und erlaubte eine orientierungsunabhängige virtuelle Schnittführung durch die erfassten Strukturen. Die Bilddaten zeigten für gesundes Pankreas, einen PanNET und ein PDAC jeweils distinkte morphologische Signaturen, die in konventionellen zweidimensionalen Schnitten nur partiell erfasst werden.

### Fallbeispiel 1: Gesundes Pankreasgewebe

**Abb. 2:**
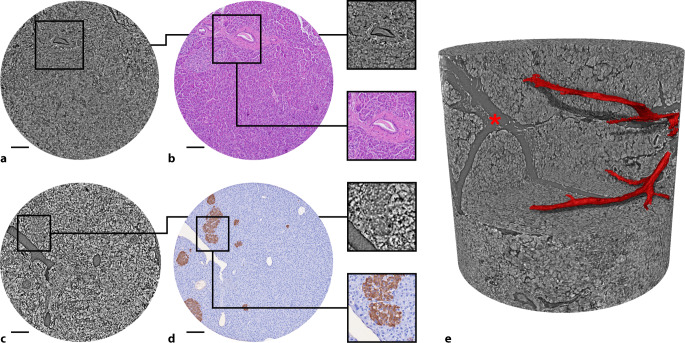
Untersuchung von gesundem Pankreasgewebe. Im SRµCT-Datensatz können duktale und vaskuläre Strukturen dargestellt (**a**) und mit einem histologischen Gewebeschnitt (HE) korreliert werden (**b**, *Vergrößerungsglas*: Pankreasgang im Querschnitt). **c** Ebenfalls können endokrine Organanteile im volumetrischen Nativbild dargestellt werden. **d** Durch Korrelation mit der immunhistochemischen Färbung gegen Insulin (*braun*) kann dies validiert werden (*Vergrößerungsglas*: Langerhans-Insel) **e** Die 3D-Rekonstruktion des Datensatzes erlaubt die Segmentierung von bspw. vaskulären Strukturen (hier: *rot angefärbt*) und deren dreidimensionale Visualisierung (*Stern*: Gewebespalt zwischen Lobuli). *Skala*: 100 µm

Als Referenzstruktur wurde gesundes Pankreasgewebe untersucht. Dieses wurde aus nichttransplantablem Pankreasgewebe aus dem Organspendeverfahren gewonnen und aus der Pankreasbiobank bezogen. Entsprechend können keine klinischen Angaben zur Verfügung gestellt werden. Das Volumen zeigte eine gut organisierte lobuläre Gewebearchitektur mit klar definierter Trennung zwischen exokrinen azinären Drüsenstrukturen und endokrinen Inseln (Abb. 2). Die Inselzellaggregate waren gleichmäßig verteilt, rundlich bis oval geformt und von einem filigranen Kapillarnetz durchzogen. Die duktalen Strukturen waren hierarchisch gegliedert, von kleinen interkalären bis hin zu interlobulären Gängen, mit glatter luminaler Konturierung und scharfer Epithelabgrenzung. Durch weiterführende Analysen kann die Verteilung von Langerhans-Inseln und die hierarchische Gliederung der vaskulären und duktalen Systeme in den 3D-Rekonstruktionen analysiert werden (z. B. Verzweigungsgrad, Tortuosität, Volumenanteile), ohne dass hierzu weitere Färbungen erforderlich sind.

### Fallbeispiel 2: Gut differenzierter neuroendokriner Tumor des Pankreas

**Abb. 3:**
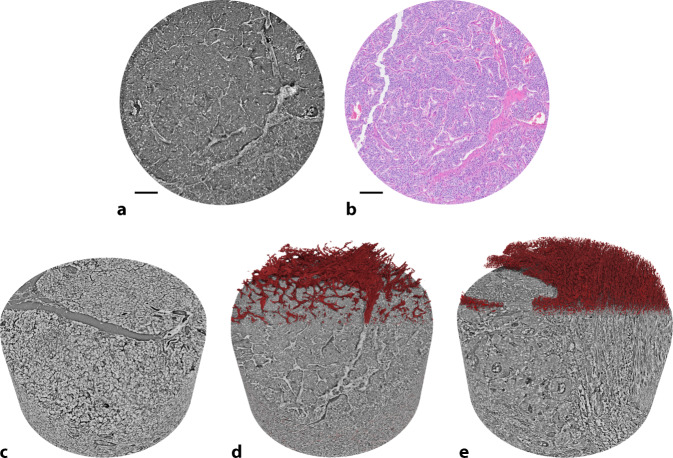
Untersuchung eines gut differenzierten neuroendokrinen Tumors (PanNET). **a** Darstellung eines 2D-Schnittes durch den SRµCT-Datensatz eines Gastrinoms. **b** Korrelation des Volumens mit klassischer HE-Färbung. Die zellulären Tumoraggregate zeigen sich von Stroma umgeben. **c**–**e** Die virtuelle Anfärbung von Bindegewebsanteilen (hier: *rot*) hebt Unterschiede der 3D-Morphologie von gesundem Pankreasgewebe im Vergleich mit pathologischen Veränderungen hervor. **c** Im gesunden Pankreasgewebe kann kein signifikanter Bindegewebsanteil erkannt werden (fehlende rot gefärbte Bindegewebsstrukturen). **d** Dagegen unterstreicht das Stromagewebe im PanNET dessen trabekuläres Wachstumsmuster. **e** Die stromalen Gewebeanteile im hier untersuchten duktalen Adenokarzinom sind heterogen verteilt und unterschieden sich morphologisch deutlich von den Septierungen des PanNET. *Skala*: 100 µm

Das untersuchte Gewebe stammt aus einem klinisch symptomatischen Gastrinom des Processus uncinatus, welches enukleiert wurde. Der solide, 2,0 × 1,5 × 1,3 cm messende Tumor zeigt in der klassischen mikroskopischen Beurteilung ein trabekuläres Wachstumsmuster mit hyperchromatischen, isomorphen Zellkernen mit deutlicher Positivität für Gastrin. Der Ki67-Index betrug < 2 %. Entsprechend pathologischer Begutachtung wurde der Befund als gut differenziertes Gastrinom, pT2, pN0 (0/1), L0, V0, G1, klassifiziert. Die zum OP-Zeitpunkt 38-jährige weibliche Patientin nimmt weiterhin an einem strukturierten Tumornachsorgeprogramm in domo teil und zeigt seit über 6 Jahren keinen Anhalt für ein Tumorrezidiv. Der PanNET war als solide aufgebautes zelluläres Aggregat erkennbar, mit relativ homogener Binnenarchitektur. In der Volumendarstellung zeigten sich trabekuläre Strukturen bei ubiquitärem Zellreichtum mit geringer Stromakomponente (Abb. 3c). Der Vergleich mit gesundem Pankreas zeigte die Abwesenheit regulärer Inselzellmuster und den Ersatz des azinären Gewebes durch relativ monomorphe Zellverbände. Die Volumendarstellung erlaubte eine detaillierte Analyse der Tumor-Stroma-Architektur (Abb. 3d), welche in 2D-Schnitten nur bedingt nachvollziehbar ist. Die 3D-Rekonstruktion und Visualisierung erleichtert den Vergleich der Stromarchitektur zwischen den hier berichteten pathologischen Gewebeveränderungen.

### Fallbeispiel 3: Duktales Adenokarzinom des Pankreas

**Abb. 4:**
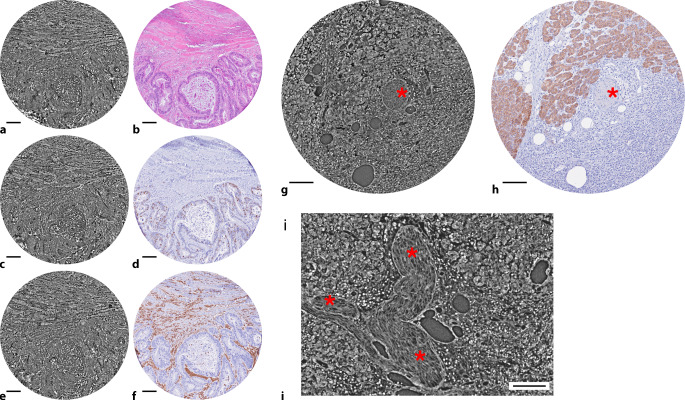
Untersuchung von Primärtumorgewebe eines neoadjuvant vorbehandelten, lokal fortgeschrittenen und hepatisch metastasierten duktalen Adenokarzinom des Pankreas. Das volumetrisch erfasste Gewebe kann mit Gewebeschnitten korreliert werden. **a** Ein virtueller Schnitt durch den SRµCT-Datensatz (**a**) korreliert mit einer HE-Färbung des Gewebeschnittes (**b**). Ein virtueller Schnitt durch den SRµCT-Datensatz (**c**) korreliert mit einer immunhistochemischen Färbung gegen Ki-67 (**d**). Ein virtueller Schnitt durch den SRµCT-Datensatz (**e**) korreliert mit einer immunhistochemischen Färbung gegen Vimentin (**f**). Ein virtueller Schnitt durch den SRµCT-Datensatz (**g**) korreliert mit einer immunhistochemischen Färbung gegen E‑Cadherin (**h**). **g**, **h** Nebenbefundlich kommt hier eine angeschnittene nervale Struktur zur Darstellung (*Stern*). **i** Die Möglichkeit der dreidimensionalen virtuellen Schnittführung erlaubt es, derartige Strukturen nachzuvollziehen. So kann die Aufzweigung der nervalen Struktur (*Stern*) in anderen, virtuellen Schnittwinkeln genau dargestellt werden. *Skala*: 100 µm

Wir berichten hier von einer zum Zeitpunkt der Operation 67-jährigen weiblichen Patientin mit lokal fortgeschrittenem und hepatisch metastasiertem duktalem Adenokarzinom des Pankreas. Die neoadjuvante Chemotherapie mit mFOLFIRINOX musste nach 2 Zyklen abgebrochen werden, woraufhin sich die Patientin mangels systemtherapeutischer Alternativen für eine operative Therapie entschied. Es wurde eine Pankreaslinksresektion mit Splenektomie, Resektion der linken Niere und Nebenniere sowie atypische Leberresektion einer singulären hepatischen Metastase durchgeführt. In der histopathologischen Beurteilung zeigte sich ein drüsiges, teils einzelliges Tumorwachstum mit typischer desmoplastischer Stromareaktion. Entsprechend pathologischer Begutachtung wurde der Befund als duktales Adenokarzinom des Pankreas, ypT3, ypN2 (5/18), ypM1 (HEP), L1, V1, Pn1, R1 klassifiziert. Der Datensatz zeigte eine heterogene, destruktive und infiltrative Gewebearchitektur. In der 3D-Darstellung waren die Tumorzellen als irreguläre, miteinander kommunizierende luminale Strukturen sichtbar. Daneben stellten sie sich teils einzeln, teils in kleinen unregelmäßigen, soliden Aggregaten liegend dar. Die Tumorzellen waren eingefasst in eine ausgeprägte Stromadesmoplasie und zeigten eine komplexe Infiltration in umgebende Gewebebereiche. Weiter zeigt sich eine nervale Struktur, welche sich entlang des Tumorgewebes zieht (Abb. 4g, h, i). Die Infiltration von Gefäß‑, Lymph- und Nervenbahnen sind bekannte pathophysiologische Eigenschaften des PDAC [6]. Bei detaillierter Untersuchung ist in nahezu allen Pankreaskarzinomgeweben eine Perineuralscheideninvasion zu erkennen [5, 17]. Diese wurde als unabhängiger prognostisch ungünstiger Risikofaktor identifiziert [5, 18]. Diese Perineuralscheideninvasion korreliert ebenfalls mit dem Risiko für ein Lokalrezidiv bzw. eine lokale Tumorpersistenz im Bereich des Nervenplexus um Truncus coeliacus und A. mesenterica superior [18]. Insbesondere die 3D-Darstellung von komplex verzweigten Strukturen (Abb. 4i) und Infiltrationsmustern waren gut darstellbar und eröffneten ein umfassendes Verständnis des Wachstums. Verschiedene histologische Stanzen aus einem Paraffinblock zeigen zudem eine deutliche Intratumorheterogenität der Morphologie des Karzinoms (vgl. Abb. 4a, g).

## Diskussion

Die vorgestellten volumetrischen Fallanalysen unterstreichen ein wissenschaftliches Potenzial der SRµCT zur differenzierten Charakterisierung pankreatischer Gewebearchitektur. Die zerstörungsfreie Messung ermöglicht die Rekonstruktion von hochauflösenden 3D-Bilddatensätzen. Die intakten Proben können für nachfolgende somatische Sequenzierungen, Massenspektrometrie, „spatial transcriptomics“ oder spezielle immunhistochemische Untersuchungen verwendet werden. Prinzipiell können Gewebe in Resin, Paraffin oder in Flüssigkeiten asserviert untersucht werden [9]. Die eingeschränkte Verfügbarkeit und Zugänglichkeit von Synchrotronen ist die größte Limitation in der breiten Anwendung dieser Technologie. Neben der hier vorgestellten Methodik bestehen andere Möglichkeiten, 3D-Geweberepräsentationen zu generieren. Durch serielle Anfertigung von Gewebeschnitten kann eine 3D-Rekonstruktion eines Volumens erreicht werden [19, 20]. Die axiale Auflösung ist dadurch auf ca. 4 µm beschränkt (Dicke des Gewebeschnittes). Daneben stehen Techniken zur optischen Gewebeklärung zur Verfügung [1]. Nachteile dieser Techniken umfassen die Notwendigkeit von immunhistochemischen Markierungen für etwaige Visualisierungen. Dies erfordert eine Permeabilisierung des Gewebes und lange Antikörper-Inkubationszeiten [1, 6]. Die Penetrationstiefe der Antikörper ist ein limitierender Faktor und gerade im Fall von stromareichem Tumorgewebe reduziert [6, 21]. Aufgrund der notwendigen Beleuchtungszeiten der nachfolgenden Lichtscheibenmikroskopie kann die Datenerhebung mehrere Stunden in Anspruch nehmen [1]. Auch eine nachfolgende Verwendung des Gewebes für weitere Untersuchungen, wie beispielsweise Sequenzierungen, wird durch die bei der Permeabilisierung verwendeten Chemikalien eingeschränkt [1].

**Abb. 5:**
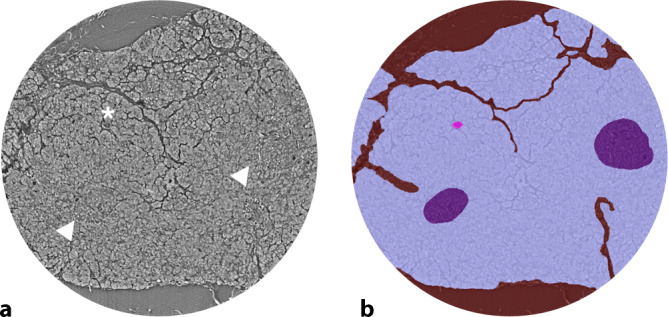
Darstellung der digitalen Annotation und Segmentierung eines volumetrischen Datensatzes. **a** Ein virtueller Schnitt durch den Datensatz zeigt exokrines Gewebe, vaskuläre Strukturen (*Stern*) und Langerhans-Inseln (*Pfeile*). **b** Diese Strukturen können manuell oder semiautomatisch annotiert (exokrines Gewebe: *hell-violett*, vaskuläre Strukturen: *pink*, Langerhans-Inseln: *dunkel-violett*) und für das Training neuronaler Netzwerke zur automatisierten Segmentierung verwendet werden

Ein besonderes Augenmerk gilt der Möglichkeit einer virtuellen Schnittführung in beliebiger Raumrichtung in den SRµCT-Daten, was perspektivisch diagnostisch als auch in der Lehre von großem Nutzen ist. In diesem Zusammenhang konnte auch die Korrelation von SRµCT mit 3D-Datensätzen anderer, unter anderem klinischer Bildgebungsmodalitäten und damit die Lokalisation des FOV über verschiedene Skalenniveaus hinweg gezeigt werden [7]. Eine Weiterentwicklung phasenkontrastbasierter 3D-Technologie ist die am ESRF etablierte hierarchische Phasenkontrasttomographie (HiP-CT) [22, 23]. Diese Methodik erlaubt die Abbildung kompletter, intakter menschlicher Organe mit sukzessive steigender Auflösung bis zu 1 µm. Solche multiskaligen Ansätze verdeutlichen, dass 3D-Bildgebung nicht nur für die Untersuchung individueller Krankheitsmuster in hoher Auflösung, sondern auch für den Aufbau organumspannender Referenzatlanten von grundlegender Bedeutung ist (Human Organ Atlas Projekt, https://human-organ-atlas.esrf.fr). Insgesamt unterstreicht unsere Untersuchung, dass die SRµCT ein innovatives Werkzeug zur dreidimensionalen Charakterisierung pankreatischer Gewebestrukturen ist. Sie ergänzt die klassische Histopathologie um eine räumlich umfassende Perspektive, ermöglicht eine differenzierte Analyse normaler und pathologischer Strukturen und legt die Grundlage für zukünftige Anwendungen in Diagnostik, Forschung und Lehre. Perspektivisch eröffnet die Kombination hochauflösender 3D-Datensätze mit KI-gestützten Segmentierungs- und Analyseverfahren die Möglichkeit, morphologische Merkmale automatisiert, reproduzierbar und quantitativ zu erfassen (Abb. 5).

## Fazit für die Praxis

- Synchrotronbasierte Phasenkontrastbildgebung ermöglicht die hochauflösende, dreidimensionale Darstellung von Weichgewebeproben ohne Färbung oder Zerstörung.
- Komplexe Gewebearchitekturen, z. B. bei Tumoren des Pankreas, lassen sich räumlich einfacher beurteilen als in konventionellen histologischen 2D-Schnitten.
- Virtuelle Schnittführung und Volumenanalyse bieten neue diagnostische und didaktische Perspektiven für Pathologie und Forschung.
- Die zerstörungsfreie Untersuchung ermöglicht nachfolgend bspw. korrelative histologische und immunhistochemische Färbung.
- Die Methode eignet sich perspektivisch für die Etablierung digitaler 3D-Gewebearchive und KI-basierter Analysen in der digitalen Pathologie.
- Eine breite Verwendung ist durch die eingeschränkte Verfügbarkeit sowie die notwendige spezifische Methodenkompetenz limitiert.

## Data Availability

Die erhobenen Datensätze können auf begründete Anfrage in anonymisierter Form beim korrespondierenden Autor angefordert werden.
